# The role of polyclonal intravenous immunoglobulin in treating HIV-infected children with severe bacterial infections: A retrospective cohort study

**DOI:** 10.1186/1471-2334-8-127

**Published:** 2008-09-24

**Authors:** Lyen C Huang, Landon Myer, Heather B Jaspan

**Affiliations:** 1 School of Public Health and Family Medicine, University of Cape Town, Faculty of Health Sciences, Anzio Road, Observatory 7925, Cape Town, South Africa; 2 Department of Epidemiology, Mailman School of Public Health, Columbia University, 722 West 168th Street, New York, NY, USA; 3 Desmond Tutu HIV Centre, Institute of Infectious Disease and Molecular Medicine, University of Cape Town, Anzio Road, Observatory 7925, Cape Town, South Africa; 4 Children's Hospital and Regional Medical Centre, University of Washington, 4800 Sandpoint Way NE, Seattle, WA 98105, USA

## Abstract

**Background:**

Mortality among HIV-infected children in developing countries remains high after serious bacterial infections despite the use of antibiotics. Intravenous immunoglobulin (IVIG) has been used as an adjuvant therapy to treat these infections, but little data exists regarding its efficacy, and previous studies have focused on IVIG as a prophylactic agent. We examined the impact of IVIG as an adjuvant therapy in reducing mortality and length of hospital stay in HIV-infected children with serious bacterial infections.

**Methods:**

This retrospective study focused on pediatric admissions at a large urban hospital between 2002 and 2006. Children between the ages of one month and nine years of age with laboratory confirmed HIV-status, serious bacterial infection, no prior exposure to IVIG, and a hospital length of stay of 5 days or more, were eligible for inclusion.

**Results:**

A total of 140 children (median age 1.2 years) met inclusion criteria; lower respiratory tract infection was diagnosed in 94 (67%) of the children, while 74 (53%) had bacterial sepsis. Fifty-four (39%) children were receiving antiretroviral therapy and 39 (28%) were receiving tuberculosis treatment. Overall 73 (52%) were treated with IVIG, with the majority (74%) of children receiving a single dose. Thirteen (9%) died during their hospital admission. In crude analysis IVIG was significantly associated with increased mortality was (Odds Ratio (OR): 5.8; 95% Confidence Interval (CI): 1.2–27.1) and this association was weakened by adjustment for other predictors of mortality (OR 4.3, 95% CI 0.7–27.9, p = 0.123). IVIG use was also associated with longer hospital stays.

**Conclusion:**

Administration of one to three doses of IVIG during the acute phase of illness does not appear to reduce mortality or the length of hospital stays in HIV-infected children with serious bacterial infections. However, the retrospective nature of this study makes confounding by indication difficult to control and further studies regarding the timing, dosing, and method of administration are required. Nonetheless the routine use of IVIG in resource-limited settings should be carefully considered given its high cost.

## Background

With 2.3 million children living with HIV worldwide in 2006 [1], the virus is a particular health threat to children in underdeveloped settings. South Africa has been heavily affected by the epidemic: in 2005, 30% of pregnant women in South Africa were HIV-infected and an estimated 240,000 children were infected from mother to child transmission. This number is expected to increase in the coming years [2], placing additional strain on an already over-burdened health system.

Infectious diseases are the leading cause of death among HIV-infected children in South Africa and many other developing countries, where as much as two-thirds of all under-5 deaths may be directly or indirectly related to HIV [3]. HIV-infected children are at significant risk of developing bacteremia and pneumonia, which are the most common causes of mortality among the children requiring hospital admission in South Africa [4-6].

The high mortality from infectious diseases in HIV-infected children despite intravenous (IV) antibiotic therapy has led to a search for adjuvant therapies such as intravenous immunoglobulin (IVIG). IVIG is an important component in treating some immunodeficiencies as well as autoimmune diseases [7]. For these diseases, immunoglobulin supplementation has several beneficial effects including primary antibody replacement, immune system modulation, suppression of auto-antibodies, and targeting of bacterial super-antigens [8,9]. Unfortunately, the theoretical benefits of IVIG administration to HIV-infected individuals have not been well-supported in the clinical setting. Two randomized controlled trials were unable to demonstrate any reduction in mortality from the prophylactic use of IVIG against serious bacterial infections, although some reduction in the number of hospitalizations and infections were seen [10,11].

The role of IVIG in therapeutic treatment of acute infections in other pediatric populations appears somewhat promising, although no studies have been done specifically in HIV-infected children. One prospective study found that therapeutic IVIG was strongly associated with shorter stays in a pediatric ward as well as decreased complications and mortality regardless of HIV status [12]. Meta-analyses have demonstrated some therapeutic benefit in treating sepsis in HIV negative neonates, but not other age groups [13,14]. In the adult HIV-negative population, there is conflicting evidence regarding the effect of therapeutic benefits of IVIG on overall mortality [15,16].

Despite the mixed nature of available data, IVIG continues to be used in conjunction with antibiotics to treat bacterial infections in some settings, particularly in HIV-infected children. However, the high cost of IVIG coupled with supply shortages due to increasing off-label use have resulted in calls for better guidelines and further research [7,17,18].

To our knowledge, no study has evaluated IVIG as adjunct treatment for serious bacterial infections in HIV-infected children, although its use for sepsis prevention has been evaluated. We analyzed the impact of therapeutic IVIG administration on clinical outcomes among HIV-infected children hospitalized for serious bacterial infections in Cape Town, South Africa.

## Methods

### Study design and population

We conducted a retrospective cohort study of all HIV-infected pediatric admissions at an urban public sector hospital in Cape Town between January 2002 and June 2006. Over 50% of all admissions to the ward are HIV-infected; the most common presenting conditions were acute gastroenteritis or lower respiratory tract infections. The referral population is a largely black African population living in rapidly growing settlements with residents of predominantly low socioeconomic status.

All children between one month and nine years of age with laboratory confirmed HIV-status, a diagnosis of bacterial sepsis, bacterial pneumonia, and/or meningitis, no prior exposure to IVIG, and a hospital length of stay of 5 days or more, were eligible for inclusion. Exclusion criteria included established indications for IVIG such as Kawasaki's disease, autoimmune hemolytic anemia, Guillain-Barré syndrome, or idiopathic thrombocytopenia.

Data were collected from paper charts and cross-referenced with pharmacy supply logs to confirm IVIG dosing. For IVIG unexposed patients, the first admission for each patient meeting inclusion criteria after January 1, 2002, was chosen for analysis. For IVIG exposed patients, the first admission where the child received IVIG was used. Subsequent admissions were not examined.

Approval for this study was obtained from the University of Cape Town Faculty of Health Sciences Research Ethics Committee.

### Measures

Basic demographic, laboratory and clinical information were abstracted from patient charts using a standardized abstraction form. Data were collected by a single individual. Data validity was checked by repeated blinded abstraction on randomly selected charts.

Electrolyte, metabolic, liver function, and hematological laboratory data was collected at four time points: admission, IVIG administration (or one week after admission for the unexposed group), one week after IVIG administration (or two weeks after admission for the unexposed group), and discharge. If data was not available on the exact date specified, lab values from the nearest date were used. CD4/8 counts and percentages were not recorded as part of this study due to the unavailability of these measures for most participants near the time of diagnosis.

Diagnoses of bacterial sepsis and bacterial pneumonia were included based on clinical findings including chest radiograph and blood cultures when available. A diagnosis of meningitis was included based on results of cerebrospinal fluid cultures collected from lumbar punctures. Protein-energy malnutrition (PEM) was used to describe clinical diagnoses of marasmus, Kwashiorkor or marasmic Kwashiorkor.

Hospital length of stay was defined as a binary outcome with a "long" stay defined as greater than the median length of stay for all patients. Mortality was considered only in the context of hospitalization. Patients who died after discharge were excluded from mortality totals since the date and cause of death could not be accurately discerned.

Antibiotics were analyzed individually and also grouped by relative breadth of organism coverage. Thus, first-line antibiotics were generally used as initial empiric coverage for sensitive organisms whereas fourth-line antibiotics were reserved for very resistant organisms or patients otherwise refractory to treatment. First-line antibiotics were defined as penicillin, ampicillin, gentamycin, ceftriaxone, and cefuroxime. Second-line antibiotics were cloxacillin, intravenous augmentin, cefazolin, and cefotaxime. Third-line antibiotics were piperacillin, piperacillin plus tazobactam, amikacin, cefipime, and ceftazadime. Fourth-line antibiotics were ertapenem, imipenem, meropenem, and vancomycin.

A modified PRISM III score was used to grade the severity of illness in eligible children. The PRISM III is a validated physiologically-based score for evaluating the risk of mortality [19]. Because not all of the data required for calculating a complete PRISM III score were available upon chart review, we developed a modified scoring system based on percentages. A PRISM III score was calculated from the available data for each patient and then divided by the maximum possible score that the patient could have received. When more than one lab value was applicable, the most severe lab values were used to reflect the greatest morbidity during the course of illness.

### Analysis

Data analysis was conducted using Stata 9.0 (Stata Corporation, College Station, Texas, United States). Data were described using means, medians and proportions; bivariate analyses employed Students' t-tests, Wilcoxon rank-sum tests, and chi-square tests, as appropriate. Multiple logistic regression models were used to identify independent predictors of IVIG exposure, mortality, and hospital length of stay. Models were constructed by sequential addition of predictors and comparison of models using the Akaike information criteria and the likelihood-ratio chi-squared value. Variables were eligible to be examined in the model if missing data on a particular covariate did not reduce the number of observations in the model by more than 10% of the total cohort size.

## Results

### Demographics

Of 247 patient records reviewed, 140 children met the inclusion criteria for the cohort. The main reasons for exclusion were lack of a qualifying diagnosis, unconfirmed HIV status, hospital length of stay less than 5 days, use of IVIG for Kawasaki's disease and hemolytic anemia. The demographic characteristics of the 140 included patients are presented in Table 1.

**Table 1:** Demographic description of 140 admissions, overall and stratified by IVIG exposure

Characteristic	Total cohort n (%)	IVIG Unexposed n (% of unexposed)	IVIG Exposed n (% of exposed)	p-value
IVIG exposure (% of total cohort)		68 (48)	73 (52)	
Gender				
Males	76 (54)	37 (55)	39 (53)	0.831
Females	65 (46)	30 (45)	34 (47)	
On HAART	54 (39)	21 (31)	33 (45)	0.092
On TB medication	39 (28)	17 (25)	22 (30)	0.496
Mortality	13 (9)	2 (3)	11 (15)	0.014
"Long" hospital stays	65 (46)	12 (18)	53 (73)	< 0.001
Age at admission (yrs)				
Median (IQR)	1.2 (0.5–2.3)	1.3 (0.6–2.5)	1.0 (0.4–2.1)	0.120
Weight at admission (kg)				
Median (IQR)	6.7 (4.7–9.5)	7.3 (5.0–10.9)	6.0 (4.6–8.3)	0.039
Temp. at admission (°C)				
Median (IQR)	37.3 (36.5–38.2)	37.4 (36.5–38.2)	37.3 (36.5–38.5)	0.812
Birth weight (kg)				
Mean (95% CI)	2.9 (2.8–3.0)	3.0 (2.8–3.2)	2.8 (2.7–3.0)	0.321
Hospital length of stay (days)				
Median (IQR)	15 (7–35)	8 (6–12)	30 (15–59)	< 0.001
Modified PRISM percentage				
Median (IQR)	11 (0–21)	6 (0–15)	14 (6–28)	0.004
Hemoglobin on admission				
Median (IQR)	9.1 (8.1–10.3)	9.3 (8.4–10.5)	8.7 (8.0–10.2)	0.202
Diagnosis				
Lower respiratory tract infection	94 (67)	50 (75)	44 (60)	0.062
Bacterial sepsis	74 (53)	24 (36)	50 (68)	< 0.001
Bacterial meningitis	4 (3)	2 (3)	2 (3)	0.931
TB	40 (29)	16 (24)	24 (33)	0.239
TB meningitis	2 (1)	1 (2)	1 (1)	0.951
Gastroenteritis	54 (39)	21 (31)	33 (45)	0.092
Urinary tract infection	15 (11)	4 (6)	11 (15)	0.082
PEM	16 (11)	7 (10)	9 (12)	0.727
Fungal sepsis	4 (3)	0 (0)	4 (5)	0.052
Failure to thrive	29 (21)	8 (12)	21 (29)	0.014
Cultures				
Any positive blood culture	85 (61)	29 (43)	56 (77)	< 0.001
Positive *E. coli *blood culture	4 (3)	2 (3)	2 (3)	0.931
Positive *S. pneumoniae *blood culture	21 (15)	10 (15)	11 (15)	0.981
Positive *C. albicans *urine culture	7 (5)	0 (0)	7 (10)	0.009
Received				
1st-line antibiotics	118 (84)	53 (79)	65 (89)	0.107
2nd-line antibiotics	42 (30)	12 (18)	30 (41)	0.003
3rd-line antibiotics	51 (36)	10 (15)	41 (56)	< 0.001
4th-line antibiotics	44 (31)	5 (7)	39 (53)	< 0.001
IV ampicillin	92 (66)	37 (55)	55 (75)	0.012
IV cefotaxime	32 (23)	6 (9)	26 (36)	< 0.001
IV vancomycin	21 (15)	2 (3)	19 (26)	< 0.001
IV piperacillin/tazobactam	21 (15)	4 (6)	17 (23)	0.004
IV fluconazole	14 (10)	0 (0)	14 (19)	< 0.001
IV amphotericin	8 (6)	0 (0)	8 (11)	0.005

In the cohort, 64 children (46%) were female, and the median age was 1.2 years old (Interquartile Range (IQR): 0.5–2.3). The median weight on admission was 6.74 kg (IQR 4.71–9.56). Only 66 subjects had birth weights recorded, with a median weight of 2.79 kg (IQR 2.77–3.02). A subset of children were on highly active antiretroviral therapy (HAART) and tuberculosis (TB) treatment (39% and 28% respectively).

### Hospital course

The median length of stay was 14.5 days (IQR 7–35). Eighty-five children (61%) had at least one positive blood culture during the hospital admission. Antibiotic use was common, with 118 (84%) receiving 1^st^-line antibiotics, 42 (30%) receiving 2^nd^-line antibiotics, 51 (36%) receiving 3^rd^-line antibiotics, and 44 (31%) receiving 4^th^-line antibiotics.

A diagnosis of lower respiratory tract infection was made in 94 (67%) children, while 74 (53%) were diagnosed with bacterial sepsis (proven or presumed). Only 4 (3%) children were found to have bacterial meningitis. More than one qualifying diagnosis for inclusion was found in 31 (22%) of the children.

Overall, 73 (52%) children were exposed to IVIG. Dosing was always 400–500 mg/kg of IVIG given over 3–4 hours of infusion. A single dose of IVIG was administered to 54 (74%) children, whereas 15 (21%) received two doses and 4 (5%) received three doses. During the total observation time, 13 (9%) children died in hospital with the remainder being discharged.

### Predicting IVIG exposure

Bivariate analysis revealed significant associations between IVIG and mortality (p = 0.014), "long" hospital stays (p < 0.001), presumed or proven bacterial sepsis (p < 0.0001), a diagnosis of failure to thrive (p = 0.014), at least one positive blood culture (p < 0.0001), and exposure to 2^nd ^-(p = 0.003), 3^rd ^-(p < 0.0001), and 4^th^-line antibiotics (p < 0.0001). Specific antibiotics were also associated with IVIG, such as intravenous ampicillin (p = 0.012), cefotaxime (p < 0.001), vancomycin (p < 0.001), and piperacillin/tazobactam (p = 0.004). Severity of illness, as measured by the modified PRISM score, was also associated with IVIG exposure (p = 0.004).

The best-fit logistic regression predicting IVIG exposure is presented in Table 2. Significant predictors were any positive blood culture regardless of organism (OR 4.3, 95% CI 1.6–11.8), a diagnosis of failure to thrive (OR 5.0, 95% CI 1.5–16.3), exposure to third-line (OR 4.0, 95% CI 1.4–11.5) and fourth-line antibiotics (OR 10.3, 95% CI 2.9–37.0), as well as intravenous ampicillin (OR 5.1, 95% CI 1.7–15.7) and cefotaxime (OR 6.2, 95% CI 1.7–22.5). The modified PRISM percentage was not a significant predictor of IVIG exposure in the multivariate analysis. Not included in the model are several variables in the analysis which were perfect predictors of IVIG exposure namely: diagnosis of fungal culture-proven sepsis, exposure to intravenous fluconazole, intravenous amphotericin, and positive urine culture for *Candida albicans*.

**Table 2:** Logistic regression model predicting IVIG exposure

Predictors	Adjusted IVIG exposure OR (95% CI)
Any positive blood culture	4.3 (1.6–11.8)
Diagnosis of failure to thrive	5.0 (1.5–16.3)
3rd-line antibiotics	4.0 (1.4–11.5)
4th-line antibiotics	10.3 (2.9–37.0)
IV ampicillin	5.1 (1.7–15.7)
IV cefotaxime	6.2 (1.7–22.5)

### Predicting mortality and hospital length of stay

The best-fit logistic regression models for predicting mortality and hospital length of stays are presented in Table 3. The unadjusted odds ratio of IVIG association with mortality was 5.8 (95% CI 1.2–27.1). However, the association became statistically non-significant after inclusion of significant predictors of mortality (OR 4.3, 95% CI 0.7–27.9). Significant predictors for mortality included exposure to intravenous vancomycin (OR 7.2, 95% CI 1.5–33.4), a diagnosis of PEM (OR 13.3, 95% CI 2.2–81.0), and a positive *E. coli *blood culture (OR 20.7, 95% CI 1.1–379.7). Being female was also significant for predicting mortality (OR 7.0, 95% CI 1.3–38.7).

**Table 3:** Logistic regression models predicting mortality and hospital length of stay

Predictors	Unadjusted Mortality OR (95% CI)	Adjusted Mortality OR (95% CI)	Unadjusted "Long" hospital stays OR (95% CI)	Adjusted "Long" hospital stays OR (95% CI)
IVIG exposure	5.8 (1.2–27.1)	4.3 (0.7–27.9)	12.1 (5.4–27.3)	7.5 (2.8–20.0)

IV vancomycin		7.2 (1.5–33.4)		
Diagnosis of PEM		13.3 (2.2–81.0)		
*E. coli *blood culture		20.7 (1.1–379.7)		
Female		7.0 (1.3–38.7)		
3rd-line antibiotics				3.5 (1.3–9.7)
4th-line antibiotics				3.4 (1.1–10.4)
Positive blood culture for *S. pneumoniae*				0.13 (0.03–0.6)

"Long" hospital stays were positively predicted by IVIG exposure (OR 7.5, 95% CI 2.8–20.0) even after adjustment for exposure to 3^rd^-line (OR 3.5, 95% CI 1.3–9.7) and 4^th^-line antibiotics (OR 3.4, 95% CI 1.1–10.4). A positive blood culture for *Streptococcus pneumoniae *(OR 0.13, 95% CI 0.03–0.6) was protective against a "long" hospital stay.

## Discussion

Our analysis suggests that one to three doses of IVIG provides only limited therapeutic benefit in acutely treating severe bacterial infections for HIV-infected children. Patients who received IVIG had longer hospital stays and a trend toward increased likelihood of mortality than those who did not, even after controlling for severity of illness and concomitant antibiotic exposure. Although a small benefit cannot be ruled out, the likelihood of a clinically significant benefit from IVIG appears to be low.

Theoretically, IVIG might have provided similar benefits in HIV-infection as seen in primary immunodeficiencies. However, differences in disease mechanism may explain why clinical outcomes have not been as significantly favorable in HIV-infection. HIV-infection in children often results in a relative hypergammaglobulinemia in comparison with adults, likely secondary to abnormal B-cell activation [20,21]. Despite the high immunoglobulin G (IgG) levels in children with HIV, measurement of these levels does not appear to be a good marker for humoral immune system functionality as these children often have a decreased response to vaccines [22-24]. This suggests that the poor antibody response is functional rather than quantitative in nature and simply elevating circulating IgG levels is insufficient to convey improved bacterial resistance in children.

Differences in study populations make comparison with other studies difficult. El-Nawawy et al. demonstrated that mortality in patients who received IVIG was halved from 56 to 28 percent [12]. Meta-analyses have shown very limited benefit of treating neonates, and even then only in lab-proven infection [13,16]. None of these studies distinguished between HIV-infected and non-infected children.

Thus far, the studies conducted on HIV-infected children have only examined the role of IVIG as prophylaxis. The NICHD trial showed increased time free of infections and decreases in serious bacterial infections and acute hospitalizations [10]. The PACTG protocol 51 showed that IVIG and zidovudine together reduced mild bacterial infections and acute hospitalizations [11]. However, the benefits of IVIG disappeared in children who also received daily cotrimoxazole. In both studies, mortality remained unchanged.

Differences in the timing and dosing of IVIG also make comparison across studies difficult. In this study, the majority of participants received only a single dose of 400–500 mg/kg IVIG. This dose was given when patients were significantly ill. Only two of the 21 trials included in the meta-analysis by Pildal and Getzsche administered IVIG in a single dose [15]. The other 19 trials administered treatment at intervals or continuously over the course of 1.5 to 6 days. Those in the study by El-Nawawy, et al., received equal doses administered during the first 3 days of admission [12]. Single doses of IVIG may be insufficient to provide protective effects, as it can take 4–6 months of infusion to reach a steady state [25]. The need to reach adequate protective serum levels is supported by the delay in treatment effect noted during the NICHD trial [26].

There may be several reasons why IVIG as well as vancomycin were associated with increased mortality. Late recognition of the severity of illness and delayed administration of IVIG could explain why treated children had longer hospital stays. In turn, long hospital stays lead to an increased risk of nosocomial infection potentially resistant to antibiotics and unaffected by IVIG.

Alternatively, many infections may have been viral or mycobacterial, thus explaining the ineffectiveness of antibiotics. Of the 13 children who died in-hospital, 4 had negative blood cultures for their entire stay. Excessive use of antibiotics may also have contributed to the increased mortality. The current practice in some settings, including this one, is that children receive a full course of antibiotics even if blood cultures are negative. This is due to concerns about the poor sensitivity of cultures. The overuse of antibiotics in turn may lead to the development of resistant organisms. A recent study in Tanzania found almost 14% of pediatric admissions had laboratory-confirmed bacterial sepsis despite two-thirds of them having received prior antibiotic treatment [27]. Prolonged exposure to broad spectrum antibiotics also increases the risk of developing fungal infections which are not easily recognized or treated.

Mortality in the study cohort was also associated with PEM. This is consistent with previous studies showing the combination of malnutrition and HIV increases the likelihood of mortality [28,29]. This suggests that preventative care and ensuring adequate nutrition may help mitigate the severity of illness and reduce mortality. However, reducing mortality from malnutrition in the context of HIV has been challenging and the interplay between various contributing factors remains unclear [30,31].

There were several limitations to this study. Retrospective studies cannot control which participants receive treatment, resulting in confounding by indication. Children who were more seriously ill and at greater risk of mortality were also those most likely to receive IVIG. Although an attempt to capture illness severity was done through a modified PRISM percentage, it may not have been an adequate indicator of severity. Nor has this modified score been validated in other studies. In addition, insufficient data on CD4 percentages and viral loads was available to take into account the level of immunosuppression. In the NICHD trial, only children with CD4 counts ≥ 200/mm^3 ^benefited from IVIG prophylaxis [10]. Conducting a prospective trial would address these limitations and allow for better subgroup analysis. Also, a future comparison between intravenous and subcutaneous administration may be warranted. Subcutaneous administration has become more commonplace due to its improved side effect profile, equivalent benefit, and improved quality of life, and in some cases lower costs [32]. Whether these benefits would be seen in resource-limited settings remains to be seen.

## Conclusion

To our knowledge, this is the first study to evaluate IVIG as adjunct treatment for bacterial sepsis in HIV-infected children. Previous studies have focused on sepsis prevention. Our findings suggest that one to three doses of IVIG during the acute phase of illness may have minimal to no therapeutic benefit in reducing mortality and is not associated with decreased length of hospital stay in HIV-infected children with serious bacterial infections. However, the retrospective nature of this study makes confounding by indication difficult to control. Further research will be necessary regarding whether different timing, dosing, and/or methods of administration may prove more beneficial. The benefits seen by other studies suggest earlier and/or longer administration of immunoglobulin may be more effective. However, in limited-resource settings, non-pharmacological improvements in disease prevention, detection, and follow-up care should also be strongly considered in reducing the significant morbidity and mortality seen in young infants and children living with HIV.

## Competing interests

The authors declare that they have no competing interests.

## Authors' contributions

LCH conducted the background literature review, data collection, statistical analysis, and writing of the paper. LM supervised the study design, statistical analysis, and revised the manuscript. HBJ conceived of the study, provided clinical expertise, and revised the manuscript. All authors read and approved the final manuscript.

## Pre-publication history

The pre-publication history for this paper can be accessed here:


